# Euglycemic Diabetic Ketoacidosis Associated with Sodium-Glucose Cotransporter 2 (SGLT2) Inhibitors: A Case Series and Clinical Reflections

**DOI:** 10.7759/cureus.96393

**Published:** 2025-11-08

**Authors:** Atqua Sultan, Mah Noor Naeem, Nasir Javed, Mahrukh Rehman, Ahmad Danial

**Affiliations:** 1 Anesthesia, Nishtar Medical University, Multan, PAK; 2 Intensive Care Unit, Buch International Hospital, Multan, CPV; 3 Pulmonary and Critical Care Medicine, DG Khan Medical College, Multan, PAK; 4 Pulmonology and Critical Care, Buch International Hospital, Multan, PAK; 5 Intensive Care Unit, Buch International Hospital, Multan, PAK; 6 Intensive Care Unit, Royal Free Hospital, London, GBR

**Keywords:** icu, low- and middle-income country (lmic), s: euglycemic diabetic ketoacidosis, sglt2 inhibitors, type 2 diabetes mellitus

## Abstract

Euglycemic diabetic ketoacidosis (EuDKA) is a rare but potentially life-threatening complication of sodium-glucose cotransporter-2 (SGLT2) inhibitor therapy. It is characterized by high anion-gap metabolic acidosis and ketonemia despite near-normal blood glucose levels, which can delay recognition and treatment. Reports from low- and middle-income countries (LMICs) remain limited.

This case series from an LMIC describes three patients with type 2 diabetes mellitus and multiple comorbidities, including hypertension, ischemic stroke, chronic kidney disease, and poliomyelitis developed EuDKA associated with SGLT2 inhibitor (SGLT2i) use. Despite blood glucose levels remaining below the typical diabetic ketoacidosis threshold, all patients presented with hallmark metabolic acidosis and ketonemia, posing diagnostic challenges exacerbated by overlapping acute illnesses and limited diagnostic resources. Diagnosis was based on clinical suspicion and laboratory confirmation through venous blood gas analysis, positive ketones, and high anion-gap metabolic acidosis. Management involved standard EuDKA protocols comprising intravenous fluids, insulin infusions, and electrolyte correction, which led to gradual resolution of acidosis and normalization of biochemical parameters. These cases emphasize the importance of maintaining vigilance and providing prompt treatment for EuDKA in complex clinical contexts, particularly within resource-constrained healthcare systems.

All three patients developed EuDKA related to SGLT2i use during acute illness but recovered fully following prompt diagnosis and standard DKA management with fluids, insulin, and electrolyte correction. These cases emphasize that early recognition, appropriate management, and patient counseling on temporary drug cessation during intercurrent illness are vital to prevent recurrence, especially in resource-limited settings.

## Introduction

Diabetic ketoacidosis (DKA) is a critical metabolic emergency predominantly resulting from absolute or relative insulin deficiency in patients with diabetes mellitus. It is characterized by the classical triad of marked hyperglycemia (serum glucose >250 mg/dL), ketonemia, and metabolic acidosis, often accompanied by significant electrolyte disturbances [1]. However, a subset of patients develops euglycemic diabetic ketoacidosis (EuDKA), in which blood glucose levels are normal or only mildly elevated (<250 mg/dL), posing diagnostic challenges due to the absence of significant hyperglycemia [2].

EuDKA has been reported in association with various clinical conditions, including prolonged fasting, pancreatitis, gastrointestinal losses, pregnancy, insulin pump therapy, and increasingly, the use of sodium-glucose cotransporter-2 inhibitors (SGLT2i). These inhibitors promote renal glucose excretion, leading to lower circulating glucose levels. SGLT2i-related DKA often demonstrates a more protracted course compared to classic DKA, with median resolution time nearly twice as long (92 vs. 35 hours) [3].

The clinical utility of SGLT2 inhibitors has expanded rapidly due to their favorable effects on glycemic control, weight reduction, and cardiovascular and renal protection [4,5,6]. Despite these benefits, the risk of EuDKA remains an important safety consideration. Clinical trial data estimate the incidence of EuDKA to be low, between 0.2 and 0.76 events per 1,000 patients-years, varying by agent and dosage [7]. Nevertheless, post-marketing data reveal increased cases, particularly triggered by acute illness or surgical stress, underscoring the necessity for clinical vigilance [8].

DKA is also associated with a hypercoagulable state that can precipitate thrombotic complications such as ischemic stroke. Conversely, acute vascular events may trigger DKA, resulting in a complex bidirectional relationship that complicates diagnosis and management [1,9].

This case series aims to describe the clinical course, diagnostic complexities, and management outcomes of EuDKA in patients on SGLT2 inhibitors in an LMIC ICU, and emphasize the importance of heightened clinical awareness.

## Case presentation

Case 1

A 45-year-old male with poorly controlled type 2 diabetes mellitus (HbA1c 10%), hypertension, and active smoking, on SGLT2i therapy, presented with altered sensorium, vomiting, and diarrhea. His admission glucose was normal, i.e., 162mg/dl, but blood and urine ketones were positive. Venous blood gas showed metabolic acidosis (pH 7.28, HCO₃⁻ 17.9 mmol/L, pCO₂ 38.8 mmHg). A hypertensive crisis, ischemic stroke, and myocardial infarction confounded the presentation. Nonetheless, the presence of ketonuria and acidosis in the context of SGLT2i use confirmed EuDKA. He was managed with intravenous fluids, insulin infusion, and electrolyte replacement, with resolution of ketosis and acidosis in 24 hours.

Case 2

A 52-year-old male with type 2 diabetes mellitus (HbA1c 8.2%) on metformin, sitagliptin, and Empagliflozin was admitted with an ischemic stroke involving the right posterior cerebral artery. Oral hypoglycemics were discontinued at admission. During hospitalization, he developed EuDKA characterized by metabolic acidosis, elevated ketones, and a random glucose of 138 mg/dL.

With no prior history of ketoacidosis, SGLT2i-associated EuDKA was considered the likely trigger, potentiated by acute stroke. He was treated with intravenous fluids, insulin infusion, dextrose supplementation, and electrolyte replacement. Acidosis resolved, and the anion gap normalized within 24 hours.

Case 3

A 41-year-old male with poorly controlled type 2 diabetes mellitus (HbA1c 11.6%) and a history of poliomyelitis presented with fever, abdominal pain, vomiting, and diarrhea after missing oral hypoglycemic doses, including an SGLT2i. He was drowsy and tachypneic (RR 38/minute). Investigations revealed metabolic acidosis (pH 7.11, HCO₃⁻ 5 mmol/L, pCO₂ 16 mmHg), positive blood ketones, and hypokalemia (K⁺ 3.0 mmol/L), with a blood glucose level of 144 mg/dL. He was managed in the ICU with intravenous fluids, insulin infusion, and potassium replacement. Acidosis resolved after 48 hours, and the patient was shifted out of the ICU with counselling on glycemic control and SGLT2i risks.

Table 1 provides a comparative summary of the three cases, outlining their baseline characteristics, precipitating factors, and laboratory findings. Despite variable comorbidities and illness severity, all patients demonstrated complete metabolic recovery with standard EuDKA management, underscoring the effectiveness of timely diagnosis and supportive care even in resource-limited settings.

**Table 1:** Summary of clinical and laboratory features of three cases of euglycemic diabetic ketoacidosis. SGLT2, sodium-glucose cotransporter-2

Feature	Case 1	Case 2	Case 3
Patient age and sex	45-year-old male	52-year-old male	41-year-old male
Diabetes control and medication	Poorly controlled type 2 diabetes (HbA1c 10%); on SGLT2 inhibitor; poor compliance	Type 2 diabetes (HbA1c 8.2%); on empagliflozin, metformin, sitagliptin; poor compliance	Poorly controlled type 2 diabetes (HbA1c 11.6%); on empagliflozin; poor compliance
Relevant comorbidities	Hypertension; smoker (1 pack-year for 15 years)	Chronic kidney disease	Poliomyelitis
Blood glucose level (mg/dL)	162	138	144
Laboratory findings	Blood ketones positive; metabolic acidosis (pH 7.28, HCO₃⁻ 16 mmol/L); Na⁺ 140 mmol/L; K⁺ 3.2 mmol/L	Metabolic acidosis (pH 7.25, HCO₃⁻ 17 mmol/L, BE -14); positive ketones	Metabolic acidosis (pH 7.11, HCO₃⁻ 5 mmol/L, PaCO₂ 16 mmHg); positive ketones; hypokalemia (K⁺ 3.0 mmol/L)

## Discussion

The clinical scenarios presented exemplify the occurrence of EuDKA in patients receiving SGLT2i under the influence of acute physiological stressors.

The first case demonstrates EuDKA concurrent with ischemic stroke and myocardial infarction in a patient with poorly controlled diabetes. The coexistence of DKA and ischemic stroke, though uncommon, is established in the literature with proposed mechanisms including systemic inflammatory responses, endothelial dysfunction, cerebral hypoperfusion, and a hypercoagulable state associated with DKA metabolic derangements [1,9].

Emerging reports corroborate the occurrence of SGLT2i-associated EuDKA during acute ischemic stroke, highlighting the complex and bidirectional nature of this relationship [10]. Timely recognition of this overlap is critical to prevent diagnostic delays and facilitate appropriate management. Further research and evidence are warranted in this context to develop protocols for screening concurrent conditions that may develop in EuDKA, thereby preventing morbidity and mortality.

The second case emphasizes that EuDKA may develop during hospitalization for acute stroke despite cessation of oral hypoglycemics, including SGLT2is. Factors such as metabolic stress, acute illness, reduced oral intake, and dehydration contribute to the risk of ketosis and acidosis. Data indicate that nearly half of reported FDA cases of SGLT2i-associated EuDKA involve such precipitating conditions [8], suggesting that hospital settings demand vigilant metabolic monitoring, even after drug discontinuation. It can be supported by the recommendations from a European review on the occurrence of perioperative diabetic ketoacidosis in patients using SGLT2is. The MHRA (March 2020) advised that ketone levels should be monitored in patients whose SGLT2i therapy is temporarily stopped due to major surgery or serious acute illness. Blood ketone measurement is preferred over urine testing. Treatment should only be resumed once ketone levels have normalized and the patient’s clinical condition has stabilized [11].

The third case illustrates how EuDKA can be precipitated by gastroenteritis and medication non-adherence, underscoring the critical role of patient education in the temporary cessation of SGLT2is during intercurrent illnesses to mitigate risk. Similar observations have been documented in prior studies, notably by Vettikkat and Paramba, confirming the need for clear guidance on medication management during acute illness, which is again in line with the recommendations from MHRA, as stated above [2].

While SGLT2is confer significant benefits across glycemic, cardiovascular, and renal outcomes [4,5,6], they carry a recognized albeit low risk of EuDKA. Clinical trials report incidence rates of 0.2 to 0.76 cases per 1,000 patient-years, but real-world experience indicates that extended duration and severity of EuDKA episodes require close clinical attention [3,7].

Additionally, a recent review summarizes the safety concerns of SGLT2i in the context of Ketoacidosis and provides us with some insights on risks vs benefits. It is imperative to develop awareness among physicians for a more cautious approach when dealing with acute illness with SGLTi [12].

Our case series reinforces the imperative for clinicians to maintain a high index of suspicion for EuDKA in diabetic patients on SGLT2is who present with unexplained metabolic acidosis, regardless of glucose levels and taking multiple drugs, including glucagon-like peptide-1 (GLP-1). Immediate ketone testing, early diagnosis, and initiation of treatment, including insulin, fluid resuscitation, and electrolyte correction essential to prevent morbidity and mortality associated with delayed recognition. Patient counseling on medication management during illness is equally important to prevent recurrence and adverse outcomes.

## Conclusions

All three patients in this series developed EuDKA associated with SGLT2i use in the setting of acute illness or metabolic stress, despite near-normal glucose levels. Each case achieved full metabolic recovery following timely diagnosis, intravenous fluid resuscitation, insulin infusion, and electrolyte replacement, with resolution of acidosis within 24-48 hours. These outcomes highlight that, even in resource-limited environments, early recognition and adherence to standard DKA management principles can lead to favorable results. Continued clinician vigilance and patient education on the temporary discontinuation of SGLT2is during acute illness remain critical to preventing recurrence and enhancing treatment safety.
